# Laparoendoscopic Single-Site Surgery for the Treatment of Benign Adnexal Disease: A Prospective Trial

**DOI:** 10.1155/2010/108258

**Published:** 2010-02-08

**Authors:** Anna Fagotti, Francesco Fanfani, Cristiano Rossitto, Francesco Marocco, Valerio Gallotta, Federico Romano, Giovanni Scambia

**Affiliations:** Division of Gynecologic Oncology, Catholic University of the Sacred Heart, 00168 Rome, Italy

## Abstract

*Background*. To validate feasibility, efficacy, and safeness of laparoscopic treatment of benign adnexal diseases through a single transumbilical access (LESS) in a prospective series of patients. *Methods*. A prospective clinical trial including 30 women has been conducted at the Division of Gynecology of Catholic University of Sacred Hearth of Rome. Patients underwent different laparoscopic procedures by LESS utilizing a multiport trocar and conventional straight laparoscopic instrumentation. Intra and perioperative outcome has been reported. *Results*. Ten mono/bilateral adnexectomies and 20 cystectomies have been performed by LESS approach. Laparoscopic procedures were completed through a single access in 28 cases (93.4%). No major intra- or postoperative complications were observed. Mean hospital stay was 1.3 days. *Conclusions*. LESS approach is feasible to treat benign adnexal disease with a very low conversion rate and no early or late complications. More clinical data are needed to confirm these advantages compared to standard laparoscopic technique.

## 1. Introduction

Laparoscopy has been demonstrated a valid approach in many gynecologic procedures with better results in terms of minimal perioperative morbidity and shorter hospital stay, with consequent improved quality of life compared to laparotomic approach [1, 2]. Despite this well-known advantages, laparoscopy still requires 0.5 to 1.5 cm long incisions and three to five ports to be performed, each working port implying with an inherent risk of bleeding, infection, concordant organ damage, hernia formation, and decreased cosmetic outcome [3]. Recently, some efforts have been made to decrease incisional morbidity related to parietal trauma and improve cosmetic results while maintaining the same standards of surgical care [4, 5]. In this context, minilaparoscopic approaches and natural orifice translumenal endoscopic surgery (NOTES) have been developed, utilizing the mouth, anus, vagina, or urethra to access through the peritoneum. Laparoendoscopic single-site surgery has encompassed recent terminology including single-port incision laparoscopic surgery (SILS) or single port access laparoscopic surgery (SPA). NOTES and LESS techniques have emerged as viable, feasible, and widely applicable minimally invasive procedures [6–8]. Until now LESS has been mainly used in urologic surgery but recent sporadic reports in the literature have hypothesized some applications in gynaecology [9–17]. 

Here we report our initial experience on the treatment of benign adnexal disease by LESS.

## 2. Materials and Methods

This is a single-institutional prospective clinical trial including patients affected by benign adnexal diseases and treated by a LESS approach, accrued between June and July 2009 at the Division of Gynecology, Catholic University of Rome. Selection criteria were: age between 10 and 70 years old; Body Mass Index (Kg/m^2^) up to 35; American Society of Anesthesiologists class score up to III; absence of actual pregnancies or acute pelvic inflammatory diseases and absence of liver or coagulative disorders. Clinical indications were: cystic adnexal masses with benign clinical features and major diameter equal or less than 8 cm; prophylactic adnexal removal in high risk patients; ectopic pregnancies. Laparoscopic procedures intended to perform by LESS approach were: ovarian cyst's enucleations, mono (MSO) or bilateral salpingo-oophorectomies (BSO), and exclusive salpingectomies.

All patients were adequately informed on the possible risks and benefits of this experimental technique and signed a written consent agreeing to undergo the described procedure, to convert the mini-invasive access to multiaccess standard laparoscopy or laparotomy if necessary, and to allow the use of their data prospectively. An Institutional Review Board approval from the Ethical Committee of the local hospital was obtained.

All patients were submitted to preoperative US examination and evaluation of Ca 125 serum levels. Data regarding personal history, age, BMI, clinical and diagnostic information regarding actual disease were anonymously collected in an electronic database at the time of recruitment. At the end of each procedure, intraoperative data as trocar introducing time, operative time, estimated blood loss (EBL), intra- and peri-operative complications, conversion to standard multi-access laparoscopy or laparotomy were registered. Three surgeons were involved in the protocol.

Long-term complications and histological findings were also entered in the electronic database. 

### 2.1. Surgical Technique

**Figure 1:**
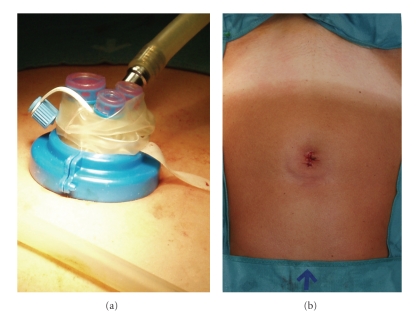
(a) Positioning of the trocar at the beginning of the procedure. (b) Postoperative umbilical scar's outcome.

Surgical procedures were performed throughout a single multiport trocar (Laparo-Endoscopic Single-Site Surgery, Olympus Winter & IBE GMBH, Hamburg, Germany), inserted in the umbilicus, as shown in Figure 1(a). The trocar is made of a doubled-over cylindrical sleeve of pliable film material which is fixed to the proximal ring and flows down around the distal ring and back up and out. To introduce the trocar, the distal ring is passed into the abdominal cavity utilizing the introducer, by an open access: a 1.5–2.0 cm longitudinal transumbilical skin incision is made, then the subcutaneous fat is opened, with exposure and consequent cold-knife incision of the abdominal fascia for approximately 1,5 cm. The parietal peritoneum is smoothly dissected with blunt scissors achieving access into the peritoneal cavity, then the introducer with the trocar distal ring is entered. Pulling on the sleeve up, the distal and the proximal ring pairs off: the procedure creates a retracting tension inside the sleeve between the rings. The valve is then positioned to fix the system, maintaining the retraction of the sleeve. This trocar is a multi-instrument access port that allows up to three laparoscopic instruments (three 5-mm cannulas or two 5-mm and one 12-mm cannula) to be used simultaneously through separate flexible channels. The cannula positions are adjustable within the flexible port, and a separate channel is available for CO2 insufflation. An intrauterine device (Uterus manipulator, Olympus Winter & IBE GMBH, Hamburg, Germany) is always utilized. 

**Figure 2:**
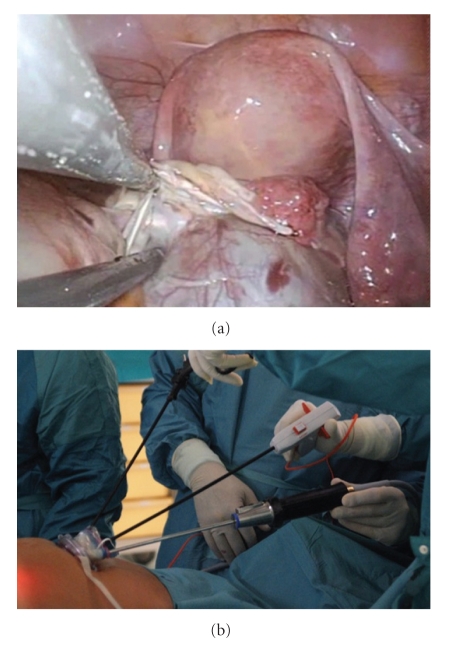
LESS operative technique: (a) internal vision, (b) external vision.

Once achieved pneumoperitoneum (12 mmHg), intra-abdominal visualization is obtained with a 5-mm 30° telescope (EndoEye, Olympus Winter & IBE GMBH, Hamburg, Germany). Working straight 5-mm instruments are inserted into the remaining 2 ports, choosing among graspers, scissors, suction/irrigation, bipolar coagulator, and a multifunctional versatile laparoscopic device which grasps coagulates and transects simultaneously (PKS Cutting Forceps, Gyrus ACMI, Hamburg, Germany) (Figure 2(b)). The combination of one standard 33 cm-long instrument with a 43 cm-long instrument is preferred in order to prevent excessive contact between surgeon's hands outside the abdominal cavity and to facilitate stripping and traction manoeuvres (Figure 2(b)). Changes in the position of the instruments and optic are carried out according to the needs of the surgeon. 

In order to perform a classic stripping for ovarian cyst enucleation by LESS approach, standard laparoscopic traction, orthogonal to the axis of the instruments (medial-lateral axis), is shifted to a parallel one (proximal-distal axis) (Figure 2(a)). Once achieved these helpful adjustments, LESS cyst enucleation results similar to standard laparoscopic procedure. For salpingo-oophorectomies the infundibulo-pelvic ligament, utero-ovarian ligament, and the tubal isthmus are grasped and coagulated with the multifunctional PKS bipolar Cutting Forceps and transacted by using the cold knife internal to the device. Specimen removal is achieved within an endo-bag inserted in the 12-mm port of the trocar.

To prevent consequent umbilical hernia formation each layer of the access port is separately sutured; in particular abdominal fascia is closed by singular stitches. Skin is repaired with rapid absorbable suture (Figure 1(b)).

## 3. Results 

Thirty women have been enrolled in the study. The following procedures have been performed by LESS: BSO/MSO (*n* = 10); mono or bilateral adnexal cyst enucleation (*n* = 20). In 2 patients (6.6%), affected by an endometriotic cyst, one 5 mm additional trocar in the iliac fossa was necessary at the end of the procedure to perform adequate haemostasis. No patient scheduled for cyst enucleation, underwent monolateral salpingo-oophorectomies due to technical limits related to the LESS approach. 

Median time to introduce the trocar from skin incision to achieved pneumoperitoneum has been 3 min (range 1–9). Port placement has been successfully executed in all cases without accidents or inadvertent port removal, but 2 patients (6.6%) showed an accidental engage of the omentum at the level of the inner ring of the trocar. This event did not hinder surgery; the omentum was released and haemostasis verified at the end of surgery throughout the hole of the trocar. No fascial, vascular, or visceral injuries, loss of pneumoperitoneum or intraoperative port-site bleeding occurred.

Rupture of the cyst was observed in 3 (2 benign ovarian tumors, 1 mature teratoma) of the 22 (13.6%) cases of cyst enucleation. We did not consider rupture of endometriotic cysts as an intraoperative adverse event, due to our surgical behavior, consisting in intentional rupture before their removal. 

Median EBL amounted to 10 mL (range 5–150). Overall median operative time was 39.5 minutes (range 18–115). According to the type of surgery, median operative time was 33 minutes (range 18–45) and 42.5 minutes (range 20–115) for BSO and ovarian cyst enucleation, respectively. This difference showed a trend to be statistically significant (*P* = .09).

No wound hematoma, wound infection, delayed bleeding, or any other postoperative complications were registered immediately after surgery.

Mean hospital stay was 1.3 days (SD: 0,5) with 86.7%, 10%, and 3.3% of the patients discharged on day 1, 2, and 3, respectively. 

No late complications were observed except for an asymptomatic 2 cm hematoma in the pelvis diagnosed by ultrasound in 1 patient (3,3%) 1 month later her cyst enucleation.

## 4. Discussion

This is a single-institutional series of patients with benign adnexal disease treated by LESS. In this series, based on simple selection criteria, in all patients considered eligible for this approach we successfully were able to complete the procedure, without conversion, early or late complications and within a reasonable operative time. Elevated BMI, previous laparotomic/laparoscopic surgery, or large cyst volume, according to our experience, do not represent a limit to perform this technique, and the introduction of the multiport trocar is simple, safe and requires progressively shorter time. 

Technical, procedural, and spatial limits related to the single access approach, reported by Ramirez as reduced visualization, loss of triangulation, and instrument interference, have been progressively minimized by some practical adjustments [17]. The evidence of lower excursion degrees among the instruments inside the abdominal cavity has been overcome by shifting the traction manoeuvre from an orthogonal axis to a parallel one whereas the use of a flexible camera on the tip did not facilitate the procedure due to its wavering when crossing the instruments [13]. Thus, in our opinion the basic surgical set for the treatment of adnexal disease by LESS should consists of a 5-mm 30° telescope, one 43-cm long, and one 33-cm long straight instruments and an intrauterine device. In fact, two different long instruments have the potential advantage to avoid crossing outside the abdominal cavity and the uterine manipulator can maintain the traction in the absence of conventional assistant's grasper. Moreover, the introduction of a multifunctional device can easily overcome the limit of a reduced number of ports. Finally, the surgical team should be composed by two surgeons, one managing both the operative instruments and the other handling the optic and moving the intrauterine manipulator, when necessary. 

The rupture rate of 13,6% in our series is analogous to data reported by previous studies, which estimated the rate of cyst's rupture during laparoscopy as being between 6 and 27% [18–20].

The only one late complication registered was diagnosed by ultrasound control we routinely get one month after surgery in patient group of this study population. She had no symptoms related to this finding.

In conclusion, our experience shows feasibility and efficacy of the LESS technique with good results in terms of adequate operative times, multiaccess low conversion rate, and limited complications showing that this approach can be safely recommended to patients affected by adnexal diseases. Larger, multicenter studies are needed to definitively confirm these preliminary results and to compare LESS technique to conventional multiaccess laparoscopy in the treatment of gynecological diseases.
